# Specific pathways mediating inflammasome activation by *Candida parapsilosis*

**DOI:** 10.1038/srep43129

**Published:** 2017-02-22

**Authors:** Adél Tóth, Erik Zajta, Katalin Csonka, Csaba Vágvölgyi, Mihai G. Netea, Attila Gácser

**Affiliations:** 1 Department of Microbiology, University of Szeged, Szeged, Hungary; 2 Department of Internal Medicine, Radboud Center for Infectious Diseases, Radboud University Medical Center, Nijmegen, The Netherlands

## Abstract

*Candida albicans* and *C. parapsilosis* are human pathogens causing severe infections. The NLRP3 inflammasome plays a crucial role in host defence against *C. albicans*, but it has been previously unknown whether *C. parapsilosis* activates this complex. Here we show that *C. parapsilosis* induces caspase-1 activation and interleukin-1β (IL-1β) secretion in THP-1, as well as primary, human macrophages. IL-1β secretion was dependent on NLRP3, K^+^-efflux, TLR4, IRAK, Syk, caspase-1, caspase-8 and NADPH-oxidase. Importantly, while *C. albicans* induced robust IL-1β release after 4 h, *C. parapsilosis* was not able to stimulate the production of IL-1β after this short incubation period. We also found that *C. parapsilosis* was phagocytosed to a lesser extent, and induced significantly lower ROS production and lysosomal cathepsin B release compared to *C. albicans*, suggesting that the low extent of inflammasome activation by *C. parapsilosis* may result from a delay in the so-called “signal 2”. In conclusion, this is the first study to examine the molecular pathways responsible for the IL-1β production in response to a non-albicans *Candida* species, and these results enhance our understanding about the immune response against *C. parapsilosis*.

Invasive fungal infections pose a serious health problem worldwide, particularly amongst immunocompromised patients^1^. *Candida* species are the most common cause of life-threatening disseminated fungal infections in critically ill patients (e.g. those who are severely immunocompromised and/or have undergone invasive procedures or major trauma)^1^. Despite the availability of antifungal drugs, the mortality associated with these infections is unacceptably high (25–40%)^2^. Although *C. albicans* remains the most common etiological agent of invasive candidiasis, the incidence of non-*albicans Candida* (NAC) spp. has been on the rise^3^. Despite this, host defence mechanisms against the NAC species is poorly understood, although evidence is emerging on the differences in immune response induced by *C. albicans* and other *Candida* spp.^4^,^5^,^6^,^7^,^8^.

*C. parapsilosis* is one of the most clinically relevant NAC species, being responsible for 17.1% of invasive *Candida* infections worldwide^9^. Newborns are especially susceptible to *C. parapsilosis* infection: 33.47% of neonatal *Candida* infections are caused by this species^10^. It is perplexing that *C. parapsilosis* causes such a large number of human infections despite being considerably less virulent than *C. albicans in vitro* and in animal models^11^,^12^. Understanding how the immune response is modulated during *C. parapsilosis* infection may explain this paradox, and ultimately lead to the development of novel, immune-based antifungal therapies.

Interleukin-1β (IL-1β) is one of the most important mediators of inflammation and acute phase response, and also contributes to the differentiation of Th17 cells, shaping adaptive immune responses^13^,^14^,^15^. In monocytes and macrophages, IL-1β is first produced in an inactive precursor form which is subsequently processed to its biologically active form by caspase-1, a component of a multiprotein complex called the inflammasome^16^. The best characterized NLRP3 inflammasome consists of NLRP3 (NLR Family, Pyrin Domain Containing 3), a two-domain adaptor protein called ASC, and pro-caspase-1^13^. The activation of the NLRP3 inflammasome is a two-step process: firstly, “signal 1” – that is typically delivered by pattern recognition receptors – initiates both *IL1B* gene transcription as well as the priming of the inflammasome by up-regulating NLRP3 mRNA and triggering post-translational changes in the NLRP3 protein, and secondly, “signal 2” induces the activation of the complex^17^. This so-called “signal 2” step of inflammasome activation can be stimulated by many different agonists, such as particulate materials, ATP or pore-forming toxins. All activators induce cationic fluxes, ROS accumulation, and/or lysosomal damage and inhibition of these cellular events prevents caspase-1 activation^17^.

The importance of inflammasome activation and IL-1β secretion during *C. albicans* infection is highlighted by the findings that mice deficient in IL-1β receptor or inflammasome components NLRP3, ASC or CASPASE1 are highly susceptible to disseminated candidiasis^14^,^18^. Furthermore, inflammasome activation triggered by *C. albicans* infection can lead to pyroptosis in macrophages. It is a proinflammatory form of programmed cell death mediated by caspase-1 activation and characterized by the loss of cell integrity^19^,^20^. Inflammasome activation by *C. albicans* is dependent on recognition by specific PRRs, such as Dectin-1 and TLR2^14^, as well as ROS production, K^+^-efflux and lysosomal rupture^21^,^22^. Importantly, inflammasome activation is also necessary for the processing of IL-18, another pro-inflammatory cytokine that plays an important role in the activation of Th1 lymphocytes^23^.

Although the role of inflammasome activation in immunity against *C. albicans* infection has been well established, little is known about its role in host defence against NAC species. *C. parapsilosis* induces the transcription of pro-IL-1β and the production of IL-1β in human macrophages^7^,^24^, but the signalling pathways leading to IL-1β secretion have not been investigated yet.

Here we used PMA-induced THP-1 as well as primary human macrophages to characterize the molecular background of IL-1β production in response to *C. parapsilosis* infection. This is the first study describing the details of inflammasome activation by a NAC species, and our results also provide new insights about the inflammatory response induced by *C. albicans*.

## Results

### *C. parapsilosis* activates the NLRP3 inflammasome in THP-1 cells and primary human macrophages

**Figure 1:**
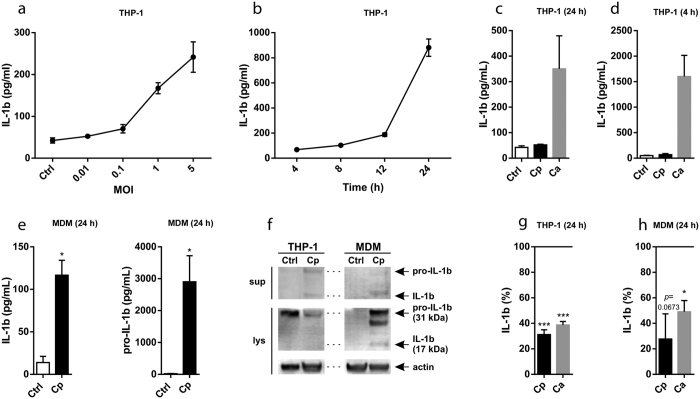
*C. parapsilosis* induces caspase-dependent IL-1β release in human macrophages. THP-1 macrophages or human MDMs were stimulated with *C. parapsilosis* or *C. albicans* and the concentration of secreted IL-1β (in cell culture supernatants) or intracellular pro-IL-β (in cell lysates) was determined by ELISA or Western blot (cropped). (**a**,**b**) THP-1 macrophages were stimulated with *C. parapsilosis* at different MOIs for 24 h (**a**) or at MOI 5 for the indicated incubation periods (**b**). (**c**,**d**) THP-1 macrophages were stimulated with *C. parapsilosis* or *C. albicans* at MOI 0.01 for 24 h (**c**) or at MOI 5 for 4 h (**d**). (**e**) MDMs were co-incubated with *C. parapsilosis* at MOI 5 for 24 h. (**f**) THP-1 macrophages or MDMs were stimulated with *C. parapsilosis* at MOI 5 for 24 h. (**g**,**h**) THP-1 macrophages (**g**) or MDMs (**h**) were stimulated with *C. parapsilosis* (MOI 5, 24 h) or *C. albicans* (MOI 0.04, 24 h) in the presence or absence of pan-caspase inhibitor Z-VAD-FMK (10 μM). IL-1β production is expressed as % of control (IL-1β secretion measured in cells stimulated with *Candida* spp + vehicle). Results (mean ± SEM) are pooled data from at least three (**a**,**c**,**d**,**e**,**g**,**h**) or representative of two (**b**,**f**) independent experiments. Ctrl, control (medium-treated cells); Ca, *C. albicans*; Cp, *C. parapsilosis*; sup, supernatants; lys, cell lysates; MOI, multiplicity of infection. *p < 0.05, ***p < 0.001 (compared to control) as determined by paired t-test.

First, we stimulated PMA-differentiated THP-1 macrophages for 24 h with different doses of *C. parapsilosis* cells and measured the amount of secreted IL-1β in cell culture supernatants. We found that live but not heat-inactivated *C. parapsilosis* induced the secretion of IL-1β in a dose-dependent manner (Fig. 1a and Fig. S1a). To determine the kinetics of IL-1β secretion, we stimulated THP-1 macrophages with live *C. parapsilosis* (MOI 5) and examined the production of IL-1β at different intervals post-infection. We found that at least a 12-hour incubation period was required for *C. parapsilosis* to induce IL-1β release (Fig. 1b). Since *C. albicans* is a potent inducer of IL-1β in THP-1 cells^25^, we also stimulated THP-1 macrophages with *C. albicans* and found that this species induced a robust IL-1β production by 4 h incubation at an MOI of 5 (Fig. 1d). Notably, even a very low amount (MOI 0.01) of *C. albicans* cells was sufficient to induce IL-1β secretion in THP-1 cells after 24 h incubation (Fig. 1c).

As a result of PMA-treatment, pro-IL-1β protein is already abundant in THP-1 cells. To assess whether *C. parapsilosis* can induce the synthesis of pro-IL-1β and the release of the mature cytokine in primary cells, we stimulated human monocyte-derived macrophages (MDMs) with *C. parapsilosis* (MOI 5) and measured their cytokine production. We found that at 24 h incubation, *C. parapsilosis* induced the production of pro-IL-1β and the release of IL-1β in MDMs as well (Fig. 1e,f). IL-18 production was also induced upon 24 h stimulation with *C. parapsilosis*, in both THP-1 cells and human MDMs (Fig. S1b,c).

As IL-1β can also be processed by alternative mechanisms, we wanted to examine whether the production of IL-1β upon *C. parapsilosis* infection is a result of caspase activation. To this end, we stimulated THP-1 and primary human macrophages with *C. parapsilosis* for 24 h in the presence or absence of the pan-caspase inhibitor Z-VAD-FMK. We found that THP-1 macrophages (Fig. 1g) and MDMs (Fig. 1h) treated with the inhibitor secreted significantly less IL-1β following stimulation with *C. parapsilosis*, while the level of intracellular pro-IL-1β remained largely unaffected (data not shown). As expected, inhibition of caspase activity also decreased the production of IL-1β in response to *C. albicans* stimulation (Fig. 1g,h).

**Figure 2:**
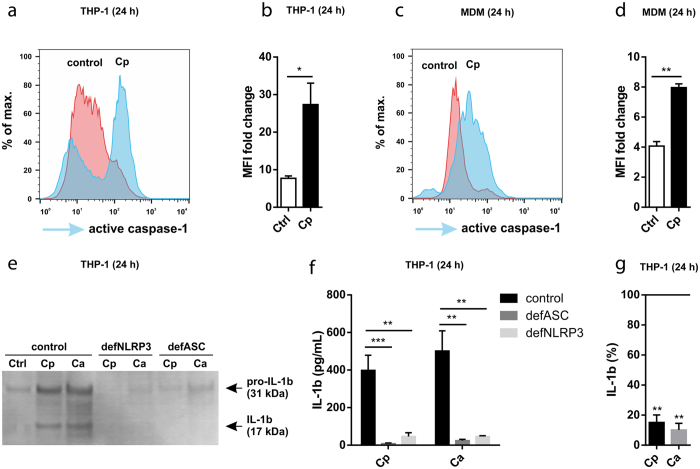
*C. parapsilosis* activates the NLRP3 inflammasome. (**a**–**d**) THP-1 macrophages (**a**,**b**) or human MDMs (**c**,**d**) were stimulated with *C. parapsilosis* (MOI 5) for 24 h and caspase-1 activation was assessed by flow cytometry using the FAM-FLICA caspase-1 assay. (**b**–**d**) Values are expressed as MFI fold change compared to unstained samples. (**e**,**f**) THP-1 macrophages (control, ASC-deficient or NLRP3-deficient) were stimulated with *C. parapsilosis* (MOI 5) or *C. albicans* (MOI 0.04) for 24 h and secreted IL-1β or pro-IL-β were determined by Western blot (cropped) (**e**) or ELISA (**f**). (**g**) THP-1 macrophages were stimulated with *C. parapsilosis* (MOI 5) or *C. albicans* (MOI 0.04) for 24 h in the presence of 50 mM KCl and the concentration of secreted IL-1β was determined by ELISA. Cytokine levels are expressed as % of control values (cytokine levels of vehicle treated samples). Results (mean ± SEM) are pooled data from (**b**,**d**,**f**,**g**) or representative of (**a**,**c**,**e**) at least three independent experiments. Ctrl, control (medium-treated cells); Ca, *C. albicans*; Cp, *C. parapsilosis*; MFI, median fluorescence intensity. *p < 0.05, **p < 0.01, *** *p* < 0.001 (compared to control) as determined by paired (**b**,**d**,**g**) or unpaired (**f**) t-test.

To examine whether *C. parapsilosis* is able to directly activate the inflammasome, we assessed the activation of caspase-1 in host cells in response to *C. parapsilosis* stimulation using a fluorescence-based assay. THP-1 macrophages as well as MDMs stimulated with *C. parapsilosis* for 24 h showed significantly increased caspase-1 activity compared to medium-control cells, indicating the activation of the inflammasome (Fig. 2a–d). As an additional control, we measured the activity of caspase-1 in *C. albicans*-stimulated cells, which showed a similar response (data not shown). To confirm that *C. parapsilosis* activates the NLRP3 inflammasome, we stimulated THP-1 macrophages deficient in inflammasome components (ASC and NLRP3) with *C. parapsilosis* cells and assessed their IL-1β production by ELISA and Western blot. IL-1β was barely detectable in these cell lines, indicating that both *C. parapsilosis* and *C. albicans* induce NLRP3 inflammasome-dependent IL-1β production (Fig. 2e,f). IL-18 production was also diminished in ASC- and NLRP3-deficient THP-1 cells upon *C. parapsilosis* or *C. albicans* stimulation (Fig. S1d).

Induction of K^+^ efflux is a requirement for NLRP3 inflammasome activation by many agonists, including *C. albicans*^21^,^25^. To examine whether decreased intracellular K^+^ concentration is also necessary for the induction of IL-1β release by *C. parapsilosis*, we examined the production of IL-1β following the blockade of K^+^-efflux by 50 mM KCl. Inhibition of K^+^-efflux dramatically decreased the secretion of IL-1β in response to *C. parapsilosis* and *C. albicans* infection (Fig. 2g). In conclusion, these results demonstrate that *C. parapsilosis* is able to induce NLRP3 inflammasome-dependent cytokine production in human macrophages.

### *C. parapsilosis*-induced IL-1β production is dependent on TLR2, TLR4, IRAK, Syk, caspase-8 and NADPH-oxidase in THP-1 macrophages

**Figure 3:**
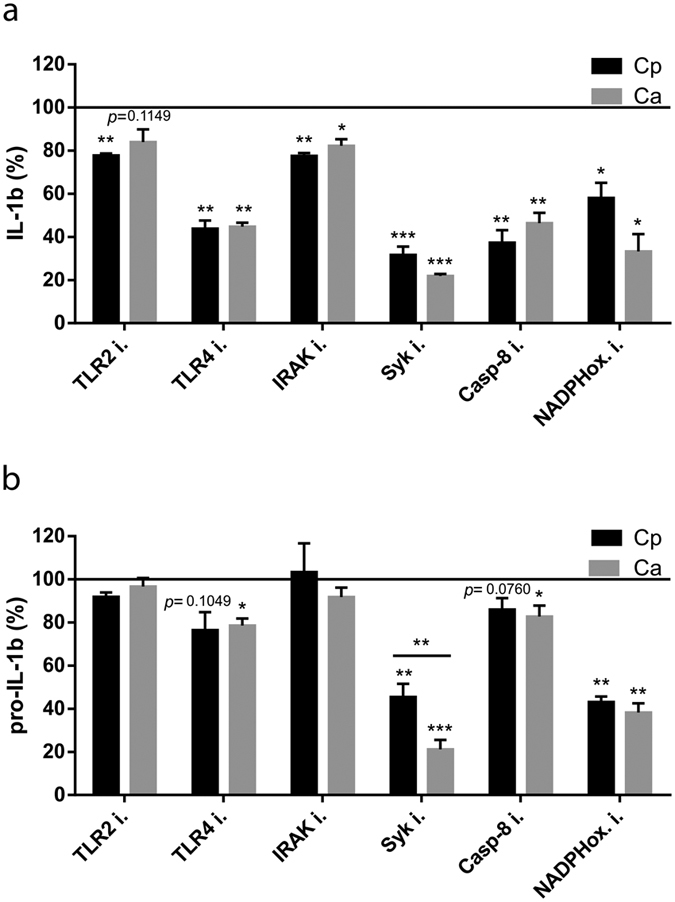
*C. paraspilosis* induces inflammasome activation by a similar mechanism as *C. albicans.* THP-1 macrophages were stimulated with *C. parapsilosis* (MOI 5) or *C. albicans* (MOI 0.04) for 24 h in the presence or absence of specific inhibitors (TLR2 mAb, 0.15 μg/ml; TLR4 inhibitor CLI-095, 5 μM; IRAK inhibitor 509093-47-4, 5 μM; Syk inhibitor ER 27319 maleate, 5 μM; caspase-8 inhibitor Z-IETD-FMK, 10 μM; NADPH-oxidase inhibitor apocynin, 600 μM). Secreted IL-1β was determined in cell culture supernatants by ELISA (**a**) and pro-IL-1β was measured by ELISA following the lysis of THP-1 cells by repeated freeze-thaw cycles (**b**). Results (mean ± SEM) are pooled data from at least three independent experiments. Ca, *C. albicans*; Cp, *C. parapsilosis*. *p < 0.05, **p < 0.01, ***p < 0.001 (compared to control) as determined by paired t-test. Cytokine levels are expressed as % of control values (cytokine levels of vehicle treated samples).

After confirming that IL-1β production induced by *C. parapsilosis* is NLRP3-dependent, we wanted to examine whether the same molecular pathways are involved in inflammasome activation upon *C. parapsilosis* and *C. albicans* stimulation. To investigate the role of TLR2, TLR4, IRAK, Syk, caspase-8 and NADPH-oxidase in *C. parapsilosis*-induced IL-1β production, we used chemical inhibitors in our experimental system, and measured the concentration of intracellular pro-IL-1β and released IL-1β by ELISA. We found that IL-1β secretion induced by *C. parapsilosis* and *C. albicans* was strongly dependent on TLR4, Syk, caspase-8 and NADPH-oxidase, while TLR2 and IRAK seemed to have a minor role in IL-1β release (Fig. 3a). While the inhibition of TLR2 and IRAK did not affect, and TLR4 as well as caspase-8 inhibition only slightly decreased the level of intracellular pro-IL-1β upon *C. parapsilosis* or *C. albicans* stimulation, blocking of Syk or NADPH-oxidase strongly reduced the amount of pro-IL-1β (Fig. 3b). Interestingly, the reduction in pro-IL-1β levels upon Syk inhibition was more profound in response to *C. albicans* infection. Taken together, these results show that TLR2, TLR4, IRAK, Syk, caspase-8 and NADPH-oxidase are involved in the induction of IL-1β production in response to both *C. albicans* and *C. parapsilosis* in THP-1 macrophages, but Syk activation may be differentially regulated upon *C. parapsilosis* and *C. albicans* infection.

### The lack of signal 2 results in delayed inflammasome activation upon *C. parapsilosis* stimulation

**Figure 4:**
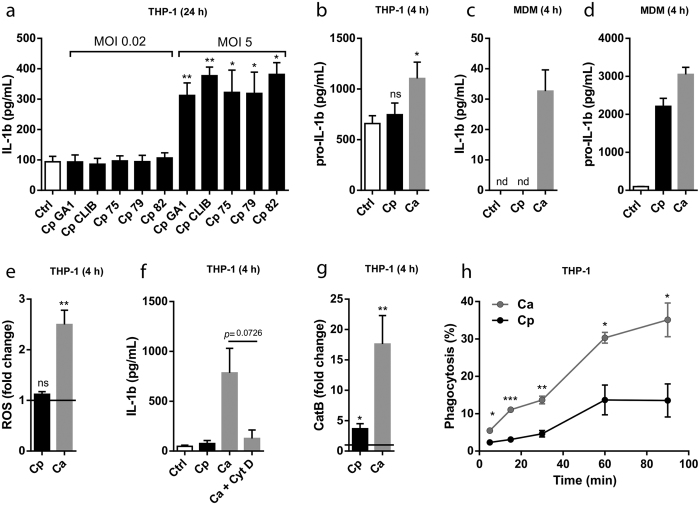
The lack of signal 2 results in delayed inflammasome activation upon *C. parapsilosis* stimulation. (**a**) THP-1 macrophages were stimulated with different *C. parapsilosis* strains at MOI 0.02 or MOI 5 for 24 h and secreted IL-1β was determined in cell culture supernatants by ELISA. (**b**) THP-1 macrophages were stimulated with *C. parapsilosis* or *C. albicans* at MOI 5 for 4 h and the amount of intracellular pro-IL-1β was measured by ELISA. (**c**,**d**) Human MDMs were stimulated with *C. parapsilosis* or *C. albicans* at MOI 5 for 4 h and the amount of secreted IL-1β or intracellular pro-IL-1β was determined by ELISA. (**e**,**g**) THP-1 macrophages were stimulated with *C. parapsilosis* or *C. albicans* at MOI 1 for 4 h. Intracellular ROS was determined by DCFDA assay (**e**), cathepsin B release was measured by a fluorometric cathepsin B activity assay kit (**g**). (**f**) THP-1 macrophages were stimulated with *C. parapsilosis* or *C. albicans* at MOI 5 for 4 h in the presence or absence of phagocytosis inhibitor cytochalasin D (2.5 μM) and the amount of secreted IL-1β was determined by ELISA. (**h**) THP-1 macrophages were infected with AlexaFluor488-labeled *C. albicans* or *C. parapsilosis* (MOI 5) for 5, 15, 30, 60 and 90 min; phagocytosis was determined by flow cytometry. Results (mean ± SEM) are pooled data from at least three (**a**,**b**,**e**–**h**) or representative of two (**c**,**d**) independent experiments. Ctrl, control (medium-treated cells); Ca, *C. albicans*; Cp, *C. parapsilosis*. *p < 0.05, **p < 0.01, ***p < 0.001 (compared to control) as determined by paired t-test.

Next, we wanted to examine why *C. parapsilosis* requires long co-incubation periods to induce inflammasome activation in host cells. As mentioned previously, *C. albicans* induced high IL-1β levels in THP-1 macrophages after 4 h (Fig. 1d). However, *C. parapsilosis* did not induce the secretion of IL-1β after 4 h incubation, even when a MOI of 10 was used (data not shown). Similarly, in contrast to *C. albicans, C. parapsilosis* could not induce the release of IL-1β at a low MOI after 24 h (Fig. 1c). To examine if this marked difference in IL-1β production is species-specific, we stimulated THP-1 macrophages with different isolates of *C. parapsilosis* and *C. albicans* for 24 h and measured their IL-1β production. We found that none of the *C. parapsilosis* strains was able to induce IL-1β release when added in a low dose (MOI 0.02), while all of them triggered similar levels of IL-1β at MOI 5 (Fig. 4a). In contrast, all of the *C. albicans* strains induced significant IL-1β production at a MOI of 0.02 (Fig. S1e).

Although pro-IL-1β is already upregulated in THP-1 macrophages following PMA-treatment, we wanted to examine whether stimulation with *Candida* spp. results in a further increase in its level. We found that while *C. parapsilosis* did not induce pro-IL-1β upregulation after 4 h, stimulation with *C. albicans* significantly increased its accumulation (Fig. 4b). On the other hand, IL-1β could not be detected in the supernatants of MDMs following 4 h stimulation with *C. parapsilosis*, despite that pro-IL-1β was significantly upregulated in these cells (Fig. 4c,d). These results suggest that although *C. parapsilosis* induces a weaker first signal than *C. albicans*, the delayed induction of the IL-1β secretion cannot be attributed solely to the inefficient production of pro-IL-1β.

Next, we wanted to examine the role of signal 2 in inflammasome activation by *C. parapsilosis*. Despite the fact that the role of ROS production in inflammasome activation is controversial, we were curious whether *C. parapsilosis* is able to induce ROS accumulation in THP-1 cells. We found that *C. parapsilosis* was not able to induce ROS generation in the first 4 hours of infection, while *C. albicans* induced a significant increase in the level of ROS (Fig. 4e). However, *C. parapsilosis* induced the generation of ROS after 12 h incubation (Fig. S1f). *C. albicans*-mediated NLRP3 inflammasome activation has also been associated with the endocytic ability of macrophages^26^. Indeed, we found that inhibition of phagocytosis with cytochalasin D diminished IL-1β production in THP-1 macrophages following 4 h stimulation with *C. albicans* (Fig. 4f). As *C. parapsilosis* does not induce the secretion of IL-1β after 4 h, and longer incubations in the presence of cytochalasin D resulted in massive cell lysis (data not shown), we could not directly assess the role of phagocytosis in *C. parapsilosis*-induced inflammasome activation. However, we evaluated the degree of phagosomal disruption by measurement of lysosomal cathepsin B release and found that *C. albicans* induced considerably higher cathepsin B release in THP-1 cells after 4 hours compared to *C. parapsilosis* (Fig. 4g). Also, we examined the kinetics of the phagocytosis of *C. parapsilosis* and *C. albicans* and found that *C. parapsilosis* cells were phagocytosed significantly more slowly by THP-1 macrophages than *C. albicans* cells (Fig. 4h). Taken together, our results suggest that the lower rate of phagocytosis and ROS production might be responsible for delayed inflammasome activation following *C. parapsilosis* stimulation.

### *C. parapsilosis* induces ASC- and NLRP3-dependent cell death in human macrophages

**Figure 5:**
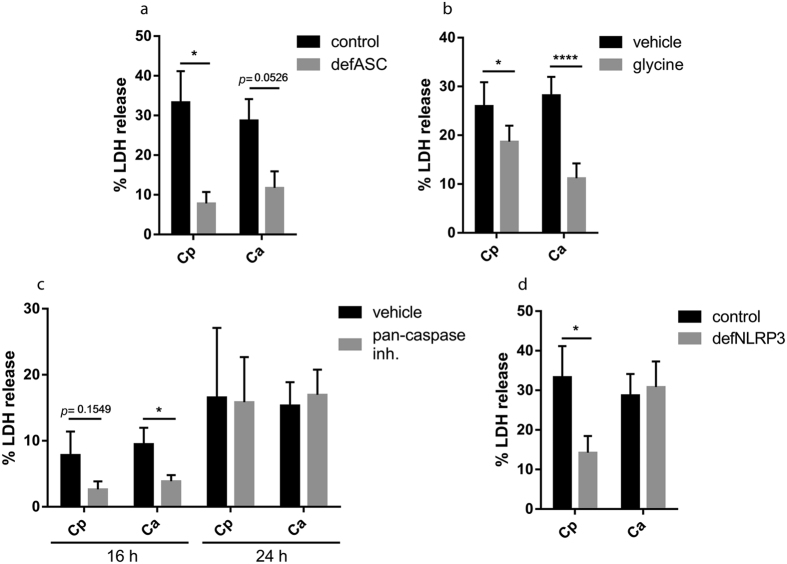
*C. parapsilosis* induces ASC- and NLRP3-dependent cell death in human macrophages. THP-1 macrophages (control as well as ASC-deficient or NLRP3-deficient, where indicated) were stimulated with *C. parapsilosis* (MOI 5) or *C. albicans* (MOI 0.04) for 24 h (**a**,**b**,**d**) or for 16 and 24 h (**c**) and cell lysis was assessed by LDH assay. LDH release is expressed as % of positive control. (**b**,**c**) THP-1 macrophages were stimulated with *Candida* spp. in the presence of pyroptosis inhibitor glycine (5 mM; **b**) or pan-caspase inhibitor Z-VAD-FMK (10 μM; **c**). Results (mean ± SEM) are pooled data from at least three independent experiments. Ca, *C. albicans*; Cp, *C. parapsilosis*. *p < 0.05, ****p < 0.0001 (compared to control) as determined by paired t-test.

As inflammasome activation is strongly connected to cell death, we also examined whether *C. parapsilosis* could induce pyroptosis in macrophages. We found that ASC deficient THP-1 macrophages showed significantly lower LDH-release upon stimulation with *C. parapsilosis* or *C. albicans* compared to control cells, indicating that both species induce ASC-dependent cell death in host cells (Fig. 5a). Also, addition of glycine reduced the amount of secreted LDH in control cells in response to both *C. parapsilosis* and *C. albicans* infection, confirming that both species induce pyroptosis in macrophages (Fig. 5b). We also examined the extent of cell lysis in the presence of pan-caspase inhibitor, and found that while it did not affect the release of LDH after 24 h incubation, the inhibitor reduced the amount of LDH after 16 h stimulation in case of both *C. parapsilosis* and *C. albicans* infection (Fig. 5c). Interestingly, however, while macrophage lysis was also reduced in NLRP3 deficient cells upon *C. parapsilosis* infection, LDH release was independent of NLRP3 in *C. albicans*-stimulated cells (Fig. 5d). These results suggest that different cell death pathways may be activated in host cells in response to *C. parapsilosis* and *C. albicans* infection.

## Discussion

In this study, we investigated the molecular mechanisms of inflammasome activation in human macrophages in response to *C. parapsilosis*. We showed that *C. parapsilosis* is able to induce inflammasome activation with subsequent IL-1β and IL-18 release in both human THP-1 macrophages and MDMs, but the induction of cytokine secretion requires a relatively long incubation period and large fungal inoculum. As a control, we also performed cell stimulations with *C. albicans* and found that a 100-fold lower dose of *C. albicans* cells was sufficient to induce IL-1β and IL-18 levels that were comparable to that induced by *C. parapsilosis*. A crucial difference between these two species is that while *C. albicans* produces hyphae, *C. parapsilosis* is only able to develop pseudohyphae^27^. It has been previously thought that hyphae formation is essential for inflammasome activation by *C. albicans*^26^; however, several non-filamenting mutants are also able to induce IL-1β production^28^. Our results confirm that the presence of true hyphae is not a prerequisite for the activation of the inflammasome by *Candida* spp. Next, we examined the molecular pathways involved in inflammasome activation induced by *C. parapsilosis*, focusing on the production of IL-1β. Generally, inflammasome priming can be initiated by any receptor that activates NFκB, such as TLRs, IL-1R or TNFR^29^. *C. albicans* can be recognized by several different PRRs that induce NFκB activation and subsequent pro-inflammatory cytokine production (reviewed in ref. 30). In macrophages, Dectin-1 and TLR2 are crucial for IL-1β synthesis in response to *C. albicans*^14^, but it is important to note that the role of PRRs in *C. albicans*-induced IL-1β production is still not completely elucidated. The PRRs mediating *C. parapsilosis*-induced IL-1β response in macrophages have not been identified yet. Here we found that in THP-1 macrophages, IL-1β production induced by either *C. parapsilosis* or *C. albicans* was dependent on TLR4. Interestingly however, pro-IL-1β accumulation was only slightly affected by TLR4 inhibition, suggesting a role for the receptor in inflammasome priming or activation rather than in the induction of pro-IL-1β synthesis. TLR4 plays an important role in the non-transcriptional priming of NLRP3^31^; it is tempting to speculate that the receptor plays a similar role in our system as well.

The tyrosine kinase Syk plays an important role in the signalling of C-type lectin receptors (such as Dectin-1, Dectin-2 or Mincle) and it is necessary for both pro-IL-1β up-regulation and caspase-1 activation in response to *C. albicans*^25^. Indeed, we found that Syk inhibition resulted in a profound reduction in pro-IL-1β and secreted IL-1β levels stimulated by *C. parapsilosis* and *C. albicans*. Interestingly, pro-IL-1β production was more strongly affected by Syk inhibition in response to *C. albicans*, suggesting a possible difference in the triggered signalling pathways leading to pro-IL-1β accumulation in *C. albicans* and *C. parapsilosis* infection.

We also found that IL-1β production was significantly reduced in THP-1 cells in the presence of caspase-8 inhibitor. Caspase-8 affects the production of IL-1β in at least two ways: on the one hand, it can be recruited to the CARD9/BCL10/MALT1 signaling complex and directly cleave pro-IL-1β upon Syk activation; on the other hand, it is required for the assembly of NLRP3 inflammasome complex and the activation of caspase-1^32^. However, we found that IL-1β production was completely abolished in NLRP3-deficient THP-1 cells, suggesting that in our experimental system, caspase-8 is required for the NLRP3-dependent, rather than the direct processing of IL-1β. At the same time, these results also indicate that inflammasome independent, alternative mechanisms^33^ do not play a role in IL-1β processing in this experimental system.

Interestingly, we found that in contrast to *C. albicans, C. parapsilosis* was not able to induce IL-1β and IL-18 production after short (4 h) incubation, even when a large inoculum was used. In theory, this could be caused by either the lack of signal 1 or signal 2 during early infection. While *C. albicans* further increased the accumulation of pro-IL-1β in THP-1 macrophages after 4 h, pro-IL-β up-regulation in response to *C. parapsilosis* was not detected in THP-1 cells at this time point, suggesting a difference in signal 1. However, stimulation with *C. parapsilosis* induced a robust increase in pro-IL-1β levels in MDMs. These results, together with the finding that *C. parapsilosis* was not able to induce the secretion of IL-1β after 4 h in THP-1 cells despite pro-IL-1β being already up-regulated suggests that either (A) *C. parapsilosis* cannot induce the post-translational modifications in NLRP3 necessary for inflammasome activation, (B) signal 2 is delayed in response to *C. parapsilosis* infection.

The role of ROS production in inflammasome activation is still a matter of intense debate. While some authors have found that inhibition of the NADPH-oxidase-dependent ROS system diminished caspase-1 activation in response to asbestos or *C. albicans*^25^,^34^, others have demonstrated that inflammasome activation is in fact increased in ROS-deficient monocytes isolated from patients with chronic granulomatous disease^35^,^36^. Here we found that *C. albicans* but not *C. parapsilosis* induced ROS accumulation in THP-1 cells after 4 hours of co-incubation. Whether this is directly associated with caspase-1 activation needs to be further investigated in the future. We also found that *C. parapsilosis* triggered significantly lower phagosomal cathepsin B release after 4 hours compared to *C. albicans*. These results suggest that the delay in IL-1β production may be caused by the lack of signal 2 during early *C. parapsilosis* infection. The requirement of phagocytosis for inflammasome activation by *C. albicans* was confirmed by our results showing that inhibition of phagocytosis by cytochalasin D abolished IL-1β production in response to *C. albicans* infection. In line with the low cathepsin B release, we found that *C. parapsilosis* was phagocytosed significantly more slowly by THP-1 macrophages than *C. albicans*. These results are in agreement with our previous findings that J774 macrophages phagocytosed *C. parapsilosis* cells much slower compared to *C. albicans*^4^. Although the specific PRRs responsible for the induction of *C. parapsilosis* phagocytosis remain to be identified, these results suggest that the differential recognition of the two *Candida* species results in different phagocytosis kinetics and, consequently, affect the extent of inflammasome activation.

Importantly, modifications in the fungal cell wall upon phagocytosis are crucial for the induction of pyroptosis by *C. albicans*^37^. As pyroptosis is strongly connected to inflammasome activation, it is likely that cell wall modifications in the phagosome influence the activation of the inflammasome as well. To investigate if *C. parapsilosis* is able to induce pyroptosis in macrophages, we assessed the LDH release of ASC-deficient THP-1 macrophages upon *C. parapsilosis* and *C. albicans* stimulation. We found that cell lysis induced by *C. parapsilosis* was significantly reduced in ASC-deficient THP-1 cells, indicating that this species is also able to induce pyroptotic cell death. Additionally, *C. parapsilosis*-induced LDH release could be inhibited by glycine, a pyroptosis inhibitor^19^. Interestingly, we also found that while *C. parapsilosis*-induced cell death was also dependent on NLRP3, *C. albicans* induced similar LDH release in both control and NLRP3-deficient cells. Although *C. albicans* induces ASC- and NLRP3-dependent pyroptosis in mouse bone marrow-derived macrophages^19^, cell death pathways induced by this pathogen in human macrophages have not been explored yet. Our results suggest that NLRP3-independent cell death pathways may also be triggered by *C. albicans*.

Besides IL-1β, IL-1α is another member of the IL-1 cytokine family that has potent pro-inflammatory functions^38^. The pro-form of the cytokine is quickly released from dying cells and can act as an alarmin^39^. IL-1α plays a non-redundant role in host defence against *C. albicans*^40^. As IL-1α can directly induce the production of pro-IL-1β^41^, we hypothesized that another reason for the delayed induction of IL-1β in response to *C. parapsilosis* might be the lack of a positive feedback loop generated by IL-1α. However, we found that IL-1α could not be detected in cell culture supernatants following the stimulation of THP-1 cells (for 4 h or 24 h) with either *C. albicans* or *C. parapsilosis* (data not shown). These results argue against the role of IL-1α in *C. albicans*- and *C. parapsilosis*-mediated inflammasome activation in THP-1 macrophages. However, IL-1α secretion was induced in human MDMs in response to both *C. parapsilosis* and *C. albicans* after 24 h (Fig. S1g); therefore, we cannot exclude the possibility that this cytokine has a role in *Candida*-induced inflammasome activation in primary macrophages.

It is a very interesting question whether *C. parapsilosis* is able to inhibit the activation of caspase-1 in host cells. Many bacterial pathogens have been shown to inhibit inflammasome activation through different mechanisms^42^, but a similar strategy in the case of fungi has not been described yet. Either *C. parapsilosis* is able to inhibit the activation of caspase-1, or the delay in IL-1β production is caused merely by the lack of signal 2, either of which may have very important *in vivo* consequences. The inflammasome plays an essential role in the induction of appropriate Th1 and Th17 responses during *C. albicans* infection *in vivo*^18^. While IL-1β is necessary for the differentiation of Th17 cells^43^, that play a central role during fungal infection by mediating neutrophil responses^22^, IL-18 enhances the IFNγ production of Th1 lymphocytes^23^. Th1 cells also play a protective role during fungal infection, by enhancing phagocytosis through the production of IFNγ^44^. We have previously shown that *C. parapsilosis* induces more IL-10, and significantly less IFNγ, IL-17 and IL-22 in human PBMCs compared to *C. albicans*, indicating a bias in Th response^5^. In light of our current findings, it is likely that the low extent of inflammasome activation is responsible for decreased Th1/Th17 differentiation during *C. parapsilosis* infection. *In vivo* experiments can confirm this hypothesis in the future. Furthermore, the low IL-1β production during *C. parapsilosis* infection may also (at least partially) explain the increased susceptibility of newborns to *C. parapsilosis* infection. It is conceivable that in immunocompetent individuals, the low level of IL-1β does not cause any problems as other effector mechanisms can keep the infection under control. However, neonates have an immature innate immune system^45^,^46^; therefore, it is possible that the lack of efficient innate defences coupled with the lack of potent Th1/Th17 responses may lead to the free proliferation of fungal cells and ultimately serious infection.

In conclusion, we have shown that *C. parapsilosis* is able to induce NLRP3 inflammasome activation in human macrophages, but only after longer incubation and when fungal cells are present in sufficiently high numbers. Although we identified several molecules that play an important role in IL-1β production stimulated by *C. parapsilosis*, the exact mechanisms of inflammasome activation are to be examined by future studies. Furthermore, *in vivo* experiments with IL-1β deficient mice in the future may provide further insight about the role of inflammasome activation in host defence during *C. parapsilosis* infection. Importantly, the results generated in this study as well as those of future projects might get us closer to the understanding of inflammatory responses during fungal infections, that may ultimately contribute to the development of more effective immune-based antifungal therapies.

## Methods

### Ethics statement

For isolation of PBMC blood was taken from healthy donors. This procedure and the respective consent documents were approved by the Institutional Human Medical Biological Research Ethics Committee of the University of Szeged. All healthy donors provided written informed consent. All experiments, were performed in accordance with guidelines and regulations of the Ethics Committee of University of Szeged, and experimental protocols were approved by this institutional committee.

### *Candida* strains

*C. parapsilosis* GA1 (SZMC 8110)^47^, CLIB214 (SZMC 1560)^48^, 75 (SZMC 8002), 79 (SZMC 1596), 82 (SZMC 8111) and *C. albicans* SC5314 (SZMC 1523)^49^, L43 (SZMC 22800) and L44 (SZMC 22801) wild type strains were used in this study. Unless otherwise indicated, experiments were performed using the *C. parapsilosis* GA1 and the *C. albicans* SC5314 wild type strains. *Candida* cells were grown overnight at 30 °C in liquid YPD medium (1% yeast extract, 2% bactopepton, and 2% glucose). Prior to experiments, cells were harvested by centrifugation, washed twice with PBS (phosphate-buffered saline; 137 mM NaCl, 2.7 mM KCl, 10 mM Na_2_HPO_4_, 2 mM KH_2_PO_4_; pH 7.4) and counted using a hemocytometer. Unless otherwise indicated, all stimulations were performed using live microorganisms. When necessary, *Candida* cells were heat killed for 30 min at 100 °C.

### Differentiation and stimulation of THP-1 macrophages

THP-1 cells (control, Sigma-Aldrich; ASC- and NLRP3-deficient, InvivoGen) were cultured in RPMI 1640 medium (Lonza) supplemented with 10% heat-inactivated FBS (Lonza), 100 U/ml penicillin (Sigma-Aldrich), 100 mg/ml streptomycin (Sigma-Aldrich) and 0.05 μM 2-mercaptoethanol. To induce macrophage differentiation, THP-1 cells were plated in 24-well tissue culture plates at a density of 5 × 10^5^ cells/well (in 500 μL medium) and treated with 10 nM phorbol myristate acetate (PMA, Sigma) for 24 h. Following PMA-treatment, cells demonstrated macrophage-like morphology, as previously described^50^. THP-1 macrophages were stimulated with *C. parapsilosis* or *C. albicans* in PMA-free medium (500 μL/well). When inhibitors were used, THP-1 macrophages were pre-incubated with the specific reagent or appropriate vehicle for 1 h (in 400 μL medium) and *Candida* cells were subsequently added in a 100 μL-volume. After the incubation period, supernatants were collected and stored at −20 °C until use.

### Differentiation and stimulation of primary MDMs

Human peripheral blood mononuclear cells were isolated from buffy coats of healthy donors using Ficoll Paque PLUS (GE Healthcare) in accordance with the instructions of the manufacturer. MDM differentiation was performed as previously described, with the exception that 24-well tissue culture plates were used^24^. Briefly, monocytes were isolated by plastic adherence and cultured in serum-free X-VIVO 15 medium (Lonza) supplemented with 100 U/mL penicillin, 100 mg/mL streptomycin and 10 ng/mL recombinant human granulocyte-macrophage colony-stimulating factor (GM-CSF, Sigma-Aldrich) for 7 days. The final density of MDM cultures was approx. 3.5 × 10^5^ cells/well. Differentiated MDMs were counted and stimulated with *C. parapsilosis* or *C. albicans* suspended in GM-CSF-free X-VIVO 15 medium (500 μL/well). In certain experiments, MDMs were pre-incubated with various inhibitors (or an appropriate vehicle) for 1 h in 400 μL medium, and 100 μL medium containing the appropriate amount of *Candida* cells was subsequently added to the wells. After the incubation period, supernatants were collected and stored at −20 °C until cytokine analysis.

### Reagents

Syk inhibitor (ER 27319 maleate), TLR2 inhibitor (human TLR2 mAb, clone 383936), isotype control for TLR2 mAb (mouse IgG2B, clone 20116), pan-caspase inhibitor (Z-VAD-FMK), caspase-8 inhibitor (Z-IETD-FMK), NADPH-oxidase inhibitor (apocynin) and phagocytosis inhibitor (cytochalasin D) were purchased from R&D Systems. TLR4 inhibitor (CLI-095) was from Invivogen, IRAK-1/4 inhibitor from EMD Millipore, glycine from Sigma-Aldrich. All inhibitors were used at concentrations indicated in the figure legends. All reagents were reconstituted in sterile dimethyl sulfoxide except for the antibodies that were dissolved in PBS and glycine that was dissolved in distilled water.

### Cytokine measurements

The concentrations of IL-1β, IL-18 and IL-1α in cell culture supernatants were determined by commercial ELISA kits (MBL for IL-18 and R&D Systems for IL-1α and - β) in accordance with the manufacturer’s instructions. The concentration of pro-IL-1β was measured by ELISA following the lysis of macrophages by repeated freeze-thaw cycles, as previously described^25^.

### Detection of caspase-1 activity by flow cytometry

Caspase-1 activity was detected by using the FAM-FLICA Caspase-1 Assay Kit (ImmunoChemistry Technologies). THP-1 macrophages or MDMs were stimulated with *C. parapsilosis* or *C. albicans* for 24 h in 12-well culture plates (10^6^ cells/well). Supernatants were removed, cells were washed with PBS, gently suspended by pipetting in 2 mL PBS, transferred to 2 mL microcentrifuge tubes and harvested by centrifugation. Subsequently, macrophages were resuspended in 200 μL RPMI 1640 medium and incubated with 30x FLICA solution (6.6 μL/sample) for 60 min at 37 °C in the dark. Following staining, cells were washed twice with 2 mL wash buffer (included in the kit) and resuspended in 300 μL wash buffer. Samples were measured using a FACSCalibur instrument (BD Biosciences) and analyzed using the FlowJo v10 software.

### Measurement of ROS production

DCFDA (2′,7′ –dichlorofluorescein diacetate) - Cellular Reactive Oxygen Species Detection Assay Kit (Abcam) was used to detect intracellular ROS production. THP-1 cell were differentiated in black, 96-well cell culture plates (10^5^ cells/well). Macrophages were washed with PBS and 25 μM DCFDA substrate was added to the samples. After incubation for 45 min at 37 °C, cells were washed with PBS, and *Candida* cells (suspended in PBS) were added to the wells (MOI 1). Control wells were treated with sterile PBS buffer only. Fluorescence was detected at 20-min intervals by a FLUOstar OPTIMA Microplate Reader (BMG Labtech). Data were expressed as fold change in fluorescence intensity relative to unstimulated cells.

### Detection of cathepsin B release

Intracellular cathepsin B activity was determined by using the Cathepsin B Activity Assay Kit (Abcam). THP-1 macrophages were stimulated with *C. parapsilosis* or *C. albicans* (MOI 1) for 4 h. Subsequently, supernatants were removed, cells were washed twice with PBS and suspended to a single cell suspension by pipetting. Cell numbers were normalized and macrophages were lysed according to the manufacturer’s instructions. Samples were centrifuged, and 50 μL supernatants were transferred into black, 96-well plates. Ac-RR-AFC substrate was added to the wells as instructed by the manufacturer and plates were incubated for 2 h at 37 °C in the dark. Fluorescence was detected by a FLUOstar OPTIMA Microplate Reader (BMG Labtech). Fluorescence intensity (correlating with the amount of cathepsin B released from lysosomes) was expressed as fold change compared to that detected in unstimulated cells.

### Phagocytosis assay

Phagocytic activity was examined as previously described^51^, with minor modifications. *Candida* cells were labelled with AlexaFluor488 carboxylic acid, succinimidyl ester (Invitrogen) and co-cultured with THP-1 macrophages at MOI 5 for 5, 15, 30, 60 or 90 min in 24-well tissue culture plates. After the incubation period, THP-1 cells were washed two times with PBS, gently suspended to a single cell suspension by pipetting, transferred to 2 mL microcentrifuge tubes, harvested by centrifugation and resuspended in 300 μL PBS. To detect the ratio of *Candida* cells adhered to host cells but not phagocytosed, experiments were also performed in the presence of 2.5 μM cytochalasin D. As the number of positive events was negligible in samples treated with cytochalasin D, we defined AlexaFluor 488^+^ macrophages as the phagocytosing cell population. Samples were measured using a FACSCalibur instrument (BD Biosciences) and analysed using the FlowJo v10 software.

### Measurement of LDH activity

LDH activity in cell culture supernatants was determined by using the LDH cytotoxicity detection kit (Takara) according to the manufacturer’s instructions. Cytotoxicity was calculated as follows: cytotoxicity (%) = (OD_experimental value_/OD_positive control_) × 100 − (OD _unstimulated control_/OD_positive control_) × 100. The supernatant of cells treated with 1% Triton X-100 served as positive control.

### Western blot

Monolayers of THP-1 (3 × 10^6^ cells/well) and primary human macrophages were infected with *Candida* in 6 well plates in 1.2 ml serum-free RPMI 1640 medium for 4 h or 24 h. Supernatants were collected and macrophages were washed twice with PBS. Subsequently, macrophages were lysed in 80 μL lysis buffer (50 mM Tris, 2 mM EDTA, 50 mM NaCl) supplemented with 2 mM phenylmethylsulfonyl fluoride (PMSF), 0.8 μL protease inhibitor cocktail and 1 mM sodium orthovanadate (all from Santa Cruz Biotechnology) by 5 freeze-thaw cycles. Cell culture supernatants from two wells per treatment were pooled and concentrated with Amicon^®^ Ultra-2 3 K Centrifugal Filter Devices. 20 μl of supernatant concentrates or cell lysates containing 20 μg of total protein were separated by NuPAGE™ Novex™ 4–12% Bis-Tris Protein Gels in 1x NuPAGE^®^ MES SDS Running Buffer and transferred onto PVDF membranes using the iBlot^®^ Dry Blotting System (Invitrogen) and respective transfer stacks. Membranes were blocked in 5% BSA-TBST (5 mM Tris, 15 mM NaCl, 0.05% Tween-20, 5% BSA, pH 7.4) for 1 h, and then washed 3 times for 5 min with TBST. Blots were incubated with rabbit polyclonal anti-IL-1β antibody (sc-7884, Santa Cruz Biotechnology, dilution 1:200) and (in the case of cell lysates) rabbit polyclonal anti-α-actin antibody (sc-1616, Santa Cruz Biotechnology, dilution 1:200) in 1% BSA-TBST overnight at 4 °C. Blots were washed 3 times for 5 min with TBST. Membranes were incubated with HRP-conjugated goat anti-rabbit IgG secondary antibody (sc-2030-R, Santa Cruz Biotechnology, dilution 1:5000) for 1 h followed by washing 3 times in TBST. Membranes were then covered with a layer of Chemiluminescence Luminol Reagent (sc-2048) for 1 min and detected with the ECL (enhanced chemiluminescence) method.

### Statistical analysis

Statistical analysis was performed using the GraphPad Prism 6 software. All experiments were performed at least twice. Paired or unpaired t-test was used to establish statistical significance (see figure legends for details) and differences between groups were considered significant at p values of <0.05.

## Additional Information

**How to cite this article**: Tóth, A. *et al*. Specific pathways mediating inflammasome activation by *Candida parapsilosis. Sci. Rep.* **7**, 43129; doi: 10.1038/srep43129 (2017).

**Publisher's note:** Springer Nature remains neutral with regard to jurisdictional claims in published maps and institutional affiliations.

## Supplementary Material

Supplementary Figure 1
