# Supplementary material for: Specific pathways mediating inflammasome activation by Candida parapsilosis
Source: Sci Rep. 2017 Feb 22;7:43129. doi: 10.1038/srep43129 (PMC5320503; doi:10.1038/srep43129)
Supplement: Supplementary Figure 1 [file srep43129-s1.pdf]

Specific pathways mediating inflammasome activation by *Candida parapsilosis*

Adél Tóth[1&], Erik Zajta[1&], Katalin Csonka[1], Csaba Vágvölgyi[1], Mihai G. Netea[2], Attila Gácser[*1]

Fig. S1. (a) THP-1 macrophages were stimulated with heat-killed *C. parapsilosis* at different MOIs for 24 h. (b-c) THP-1 macrophages (b) or MDMs (c) were co-incubated with *C. parapsilosis* (MOI 5) for 24 h. (d) THP-1 macrophages (control, ASC-deficient or NLRP3-deficient) were stimulated with *C. parapsilosis* (MOI 5) or *C. albicans* (MOI 0.04) for 24 h. (e) THP-1 macrophages were stimulated with different *C. albicans* strains at MOI 0.02 for 24 h. (f) THP-1 macrophages were stimulated with *C. parapsilosis* at MOI 5 for 12 h. (g) MDMs were stimulated with *C. parapsilosis* (MOI 5) or *C. albicans* (MOI 0.04) for 24 h. Cytokine (IL-1β, IL-18 or IL-1α) production was determined by ELISA (a-e, g), intracellular ROS was determined by DCFDA assay (f). Results (mean ± SEM) are pooled data from (a,b,d,e,f,g) or representative of (c) at least three independent experiments. Ctrl, control (medium-treated cells); Ca, *C. albicans*; Cp, *C. parapsilosis*; * $p<0.05$ (compared to control) as determined by paired (b,e,f) or unpaired (d) t-test.

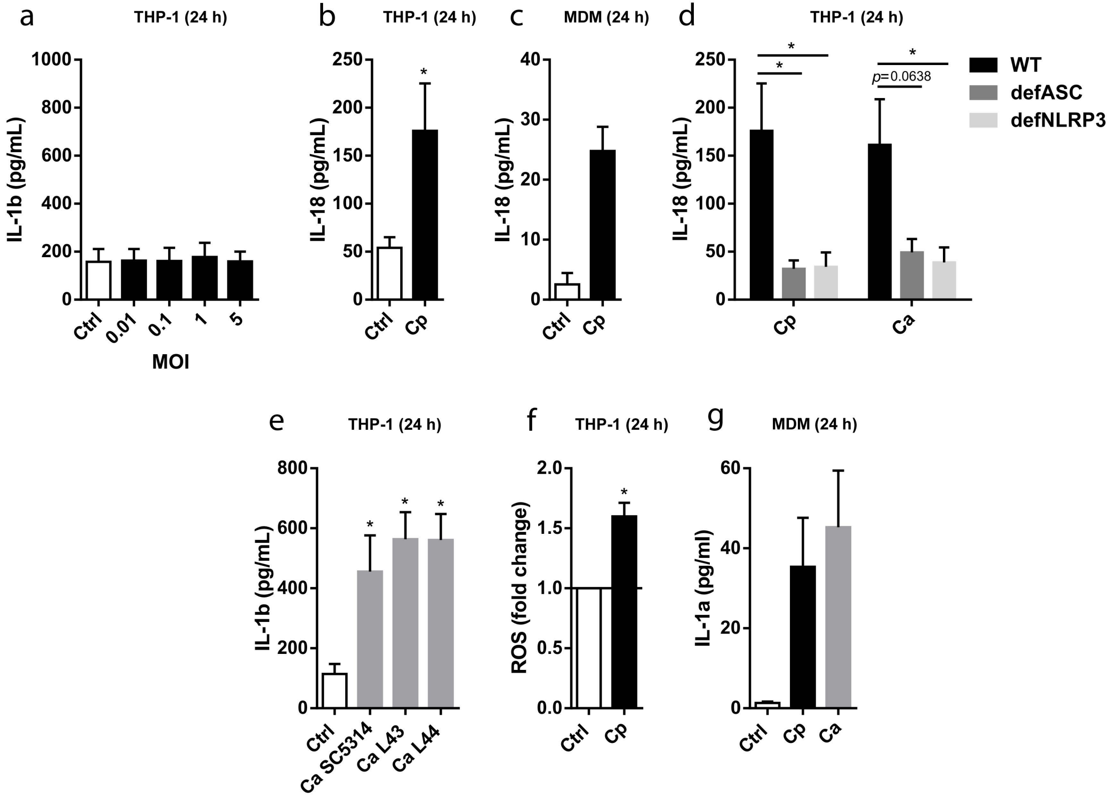
a
THP-1 (24 h)
IL-1b (pg/mL)
1000
800
600
400
200
0
Ctrl
0.01
0.1
1
5
MOI
b
THP-1 (24 h)
IL-18 (pg/mL)
250
200
150
100
50
0
*
Ctrl
Cp
c
MDM (24 h)
IL-18 (pg/mL)
40
30
20
10
0
Ctrl
Cp
d
THP-1 (24 h)
IL-18 (pg/mL)
250
200
150
100
50
0
*
*
*
p=0.0638
Cp
Ca
WT
defASC
defNLRP3
e
THP-1 (24 h)
IL-1b (pg/mL)
800
600
400
200
0
*
*
*
Ctrl
Ca SC5314
Ca L43
Ca L44
f
THP-1 (24 h)
ROS (fold change)
2.0
1.5
1.0
0.5
0.0
*
Ctrl
Cp
g
MDM (24 h)
IL-1a (pg/ml)
60
40
20
0
Ctrl
Cp
Ca
